# Adaptive Drive as a Control Strategy for Fast Scanning in Dynamic Mode Atomic Force Microscopy

**DOI:** 10.3390/s25030860

**Published:** 2025-01-31

**Authors:** Matilde Gelli, Bruno Tiribilli, Faiza Abdul Salam, Massimo Vassalli, Michele Basso

**Affiliations:** 1 Department of Information Engineering, University of Florence, 50139 Florence, Italy; michele.basso@unifi.it; 2 Institute for Complex Systems, National Research Council (ISC-CNR), 50019 Sesto Fiorentino, Italy; bruno.tiribilli@isc.cnr.it; 3 James Watt School of Engineering, University of Glasgow, Glasgow G12 8QQ, UK; faiza.salam@glasgow.ac.uk (F.A.S.); massimo.vassalli@glasgow.ac.uk (M.V.)

**Keywords:** Dynamic Mode Atomic Force Microscope, parachuting artifacts, adaptive cantilever excitation

## Abstract

Atomic Force Microscopy (AFM) is an advanced imaging technique which features nanoscale resolution and the ability to work under physiological conditions on soft samples. Modern AFM systems offer easy access to Dynamic Mode imaging which reduces the tip–sample interaction and increases the effective resolution. However, the intrinsic nature of this driving strategy induces a trade-off between three different aspects: the scanning speed, an accurate topography reconstruction and weak interaction forces. The impact of this inherent trade-off is especially evident when imaging samples with steep and deep valleys, and artifacts are often created in the reconstructed topography. This phenomenon, known as parachuting, rapidly worsens at faster speeds. In this paper, a new strategy is proposed for limiting parachuting artifacts, based on an adaptive driving strategy, which can be easily implemented as an add-on to commercial AFM systems. The suggested method has been tested on grid samples, and it enhances the nano-imaging quality by effectively reducing artifacts in the topography.

## 1. Introduction

Atomic Force Microscopy (AFM) is a high-resolution tool used for imaging and exploring material properties of substrates at the atomic level [1]. Since its invention in 1986 [2], AFM evolved to become the de facto standard for nanoscale imaging [3] and force spectroscopy characterization [4], with a wide set of non-conventional applications in the area of sensing [5,6,7]. In particular, AFM micro-cantilevers are extensively utilized as efficient micro- and nano-sensors in medical diagnostics, environmental monitoring, and industrial processes.

Over its many years of development, several modes of operation have been developed exploiting the dynamical features of the AFM cantilevers. The two most popular imaging modalities are the original Static Mode (SM) and the more modern Dynamic Mode (DM) [8]. In SM, also called Contact Mode, the probe is kept in continuous contact with the sample. Although this approach is very simple and effective, it becomes too invasive for soft samples, such as biological ones, since the continuous interaction between the tip and the sample may damage the sample, or cause an excessive wear of the tip. These limitations are overcome with DM, in which the probe oscillates, through some mechanism, near its resonance frequency. This modality is also known as Amplitude Modulation (AM) or Tapping Mode, since the cantilever taps periodically on the sample, and it not only provides the benefit of less wear, but it also allows a better accuracy [9].

This paper focuses on DM for imaging applications, and it addresses the well-known undesired phenomenon called “parachuting”, particularly evident in DM imaging, due to inherent slow dynamical responses of the AFM. In fact, parachuting occurs when steep falling edges are present in the sample, where the cantilever takes off the cliff during the scanning, losing contact with the sample, and leading to artifacts in the reconstructed image [10]. This effect worsens at higher speeds, hence defining a limit on the maximum achievable AFM scanning frequency [11].

Over the years, some techniques have been proposed to limit the impact of parachuting in fast AFM imaging. For instance, in [10], a dynamical PID controller is suggested, where the gains of the controller are increased, when required, to minimize the parachuting artifacts. This strategy has promising results; however, it may lead to an unstable configuration for higher gains, and hence, other solutions have been provided. For example, in [12], authors suggest an adaptive scan speed controller to mitigate the effects of parachuting. The algorithm allows good results and a reduced overall scanning time, but it is not reproducible on commercial AFMs since users typically cannot dynamically adapt the scanning speed.

In this work, by taking inspiration from [13], a new effective approach—called Adaptive Drive—for reducing parachuting is presented. The technique, specifically designed to be easily implemented on commercial AFMs, not only shows good performances, but it also allows users to reach higher scanning frequencies.

The paper is organized as follows. In Section 2, a proper model of the system is provided, along with a mathematical formulation of parachuting. In Section 3, the new strategy for reducing parachuting is introduced, and some details about its implementation will be addressed as well. In Section 4, some experimental data are shown, and a comparison with the traditional DM mode is discussed. Finally, in Section 5 a brief summary of the main points of the paper and future developments are tackled.

## 2. Model

**Figure 1:**
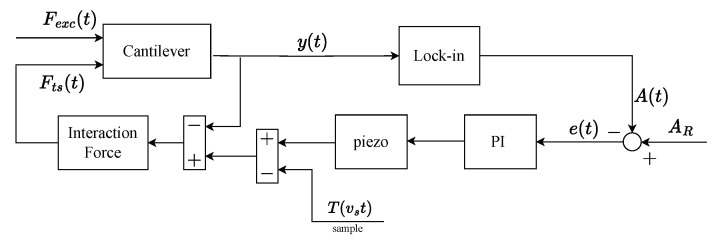
Block diagram of a standard DM-AFM setup.

The typical setup of an AFM driven in DM is shown in Figure 1.

In its simplest form, the dynamics of a DM-AFM cantilever can be described as a second-order harmonic oscillator (i.e., as a point–mass spring system) with one degree of freedom represented by the deflection y(t) [14]:

(1) $$m\ddot{y}\left(t\right)+c\dot{y}\left(t\right)+ky\left(t\right)=F_{exc}\left(t\right)+F_{ts}\left(t\right)$$

where m,c,k are effective parameters that depend on the geometry and material properties of the cantilever, while $F_{ts}\left(t\right)$ and $F_{exc}\left(t\right)$ are the tip–sample interaction force and the external drive, respectively.

In most commercial implementations, the AFM in dynamic mode is driven by a harmonic excitation

(2) $$F_{exc}\left(t\right)=F_{0}\sin\omega t$$

with a frequency close to the resonance frequency of the cantilever beam $\omega ≃\omega_{0}=\sqrt{k/m}$, and a free vibration amplitude $A_{0}$. Nevertheless, due to the interaction with the sample, the oscillation amplitude is modulated in time, resulting in a time-varying value A(t) rather than a constant [11]. However, it is desirable to keep A(t) constant during the scanning so that the sample topography can be properly reconstructed. Indeed, assuming *x* and *y*, the fast and slow scanning axes, respectively, the sample topography T(x,y) affects the AFM block diagram as a disturbance signal depending on the scanning velocity $v_{s}$ along the *x* axis, that is, T(x)=T(x,y), with $x=v_{s}t$ and *y* constant. Therefore, the control objective is to reject the disturbance signal exploiting a Proportional–Integral (PI) controller which is driven by the error signal

(3) $$e\left(t\right)=A_{R}-A\left(t\right),$$

where $A_{R}$—typically a percentage of $A_{0}$—is a suitable set-point amplitude value. The controller output is then used to translate vertically either the sample stage or the cantilever support through a piezo actuator. Ultimately, the piezo output signal u(t) can be directly used to infer the sample topography $T_{r}\left(x\right)$ along the considered scanning row, such that $T_{r}\left(x\right)=u\left(t\right)$, with $t=x/v_{s}$.

Choosing the right $A_{R}$ is the result of a tradeoff; a value very close to $A_{0}$ reduces the interaction forces with the sample, which is desirable, especially when working with soft samples. However, it reduces the input signal (e(t)) to the control, making the feedback action too weak. Usually, a good compromise is to set $A_{R}$ around 80% of $A_{0}$, resulting in good performance at standard speed for most samples. However, when the sample topography shows pronounced valleys, the major inherent limitation of such a standard approach starts to be visible. In fact, when descending over a cliff, the tip disengages from the sample and A(t) saturates to $A_{0}$, the maximum value possible when the sample is not sensed. This in turn leads the error signal e(t) to saturate to the constant value $e_{sat}=A_{R}-A_{0}$. This difference $e_{sat}$ turns out to be very small, since $A_{R}$ is close to $A_{0}$, regardless of the real separation distance between the tip and the sample. In this condition, the PI controller reacts slowly, leading to a slow descending movement of the probe towards the bottom of the valley, which in turn causes artifacts in the reconstructed topography. Such a phenomenon is known as parachuting [10], which highly affects the quality of images, and it also limits the maximum achievable scanning speed.

In [13], the authors provide a deep analysis of the parachuting effect that will be reviewed hereafter. For convenience, the piezo is approximated as a pure gain $K_{a}$, which converts the PI command into an effective movement in nanometers. Suppose we have a sample with a steep descending edge of height $h_{0}$ so that $h_{0}>>A_{0}-A_{R}$. Assuming that the tip encounters the slope at $t=t_{0}$, then parachuting occurs since the “contact” cannot be maintained. Hence, the error e(t) in input to the PI controller (see Figure 1) will converge to $e_{sat}$, and the response of the PI-piezo subsystem becomes:

(4) $$u\left(t\right)=u_{0}+K_{p}K_{a}e_{sat}+K_{i}K_{a}e_{sat}\left(t-t_{0}\right)$$

where $u_{0}$ represents the output of the subsystem before parachuting, i.e., $t<t_{0}$. However, since $e_{sat}\left(t\right)$ is very small, the contribution of the P-controller is negligible; thus, Equation (4) can be simplified as

(5) $$u\left(t\right)=u_{0}+K_{i}K_{a}e_{sat}\left(t-t_{0}\right).$$

Equation (5) defines a linear trajectory with a slope given by the product $K_{i}K_{a}e_{sat}$, and the contact with the sample will be established again only at $t_{1}=h_{0}/\left(K_{i}K_{a}e_{sat}\right)+t_{0}$, once it has passed all the way through. From this, the error in the topography $\varepsilon_{t}$ can be estimated as the area of the triangle of height $h_{0}$ and base $v_{s}\left(t_{1}-t_{0}\right)$

(6) $$\varepsilon_{t}=v_{s}\frac{h_{0}^{2}}{2K_{i}K_{a}e_{sat}}.$$

From Equation (6), it is clear that a higher $v_{s}$ leads to larger artifacts, confirming that parachuting sets a limit to the usable scanning speed.

**Figure 2:**
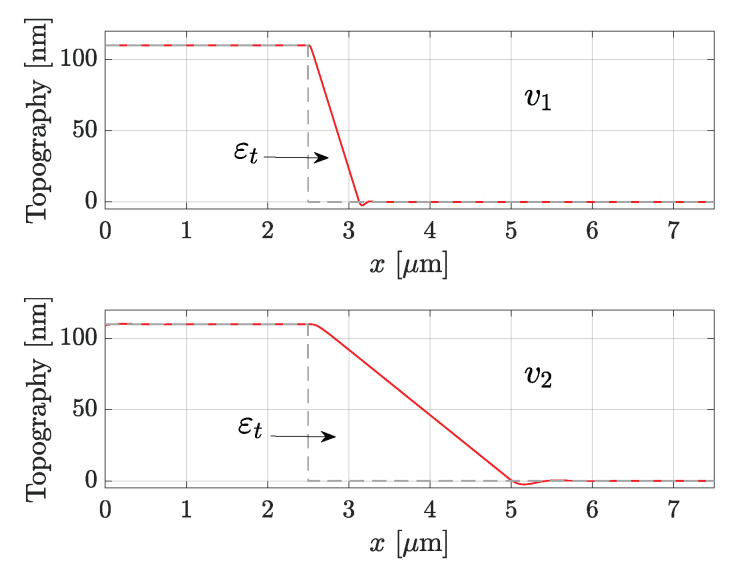
Representation of parachuting at two scanning speeds $v_{1}$ and $v_{2}$ with $v_{2}>v_{1}$, where the gray dotted curve is the reference topography T(x), and the red curve the reconstructed topography $T_{r}\left(x\right)$. Error $\varepsilon_{t}$, given by the difference between the subtended areas of $T_{r}\left(x\right)$ and T(x), turns out to be larger for a faster speed.

A DM-AFM described by Equations (1) and (2) can be easily numerically simulated, and the parachuting effect can be reproduced by providing a square wave sample as the reference topography. For example, in Figure 2, the parachuting effect was simulated at two scanning speeds $v_{1}$ and $v_{2}>v_{1}$. The gray dashed line is the reference topography T(x), while the red line is the simulated AFM response, i.e., the reconstructed topography $T_{r}\left(x\right)$. Obviously, higher scanning speeds cause a worsening of the overall performances, as the system bandwidth may not be fast enough to track the sample, but Figure 2 shows well how $v_{s}$ impacts $\varepsilon_{t}$. In fact, a higher speed quickly causes a deterioration of the reconstructed topography.

Equation (6) represents a central point, since it tells what affects parachuting. Specifically, parachuting becomes worse not only with speed, but also when the sample shows deeper valleys, since $\varepsilon_{t}$ increases with the square of the height $h_{0}$. On the contrary, a higher $K_{i}$ gain or a higher saturation value $e_{sat}$ lead to a reduction in parachuting, with the risk of an unstable configuration or too strong interaction forces, respectively.

Equation (6) hence defines a compromise among specifications that users usually have to deal with, and which is not easy to solve.

## 3. Proposed Algorithm

The major challenge for AFM manufacturers, since their invention, has been to increase the scanning speed, with the aim to reach almost video-rate imaging [3]. The reason is obvious. Faster speeds reduce the acquisition time and hence allow scanning more samples in less time. Nevertheless, from the very first attempts to achieve fast-AFM, the issue of parachuting has been recognized as one of the main limiting factors, as proved by Equation (6). However, Equation (6) is the starting point for developing alternatives techniques with respect to standard DM-AFM for reducing parachuting, and hence allowing faster, but at the same time accurate, scannings.

A first solution to parachuting was proposed by [10], which consisted in a “Dynamic-PI” controller where the P and I gains of the feedback were increased in response to parachuting, which is equivalent to provide a higher saturation value $e_{sat}$. This action reduces error $\varepsilon_{t}$, since it shortens the response time of the controller and hence the slope, but it may lead to an unstable configuration. Furthermore, the implementation of the Dynamic-PI controller requires a profound knowledge of the specific AFM device, and the possibility to modify the controller, limiting the adoption of this technique to standard AFM architectures where the controller is actually not accessible.

An alternative strategy was suggested in [12], where the authors propose an “Adaptive AFM scan speed” controller in which the speed $v_{s}$ is dynamically adapted to better follow the topography. For instance, those flat regions, where barely any important features have to be imaged, are scanned fast, while it is reduced on those areas with steep edges (either protrusion or hollows) to let the controller more efficiently track the surface. Results were provided over a grid calibration sample, and proved to be very effective since artifacts were notably reduced, but it also reduces the overall scanning speed. However, this technique—the Adaptive Speed controller—requires a modified architecture for changing the speed in real time, but clearly it cannot be reproduced on any commercial AFMs, as they do not allow such speed control.

Over the years, other techniques have been suggested based on post-processing of the acquired images [15], but they are time-consuming, and they do not overcome the issue of achieving fast-AFM. Nevertheless, in [13], an effective and easily implementable strategy was proposed. Analogously to [10], here the authors suggest a solution that speeds up the controller response by feeding a higher error signal. However, in this case, the feedback gains are not amplified, but only the deflection y(t) of the cantilever is allowed to reach a larger free vibration amplitude. The strategy was tested over either metallic and biological samples, such as insulin fibers and bacteria, and the outcomes confirmed the goodness of the strategy. Nevertheless, the algorithm was affected by some limitations, mainly related to the available electronics at the time when the paper was published. In fact, the algorithm was implemented over a Linux Real Time system and the cantilever was excited using a dither piezo, which causes a forest of peaks and the system becomes dynamically unstable, requiring an external filter set.

In this paper, the concept proposed in [13] is revisited with a few relevant modifications. Firstly, in [13], the algorithm was specifically designed for “Auto-Tapping” (AT) AFM [11,16], i.e., the cantilever is actually self-excited by exploiting an additional feedback loop, and no external driver is actually employed. This kind of modality is particularly beneficial for viscous environments; however, most AFM systems do not provide such a setting. Hence, the strategy was reconsidered for DM-AFM. Secondly, thanks to technological progress, it is now possible to implement and test the algorithm on FPGAs, which allow a faster and a more stable response. Lastly, in recent years, a new technique for driving the cantilever in oscillation was developed based on the photo-thermal excitation [17,18]. Here, the probe is excited by exploiting a laser beam focused on the free end of the cantilever; hence, it produces a much cleaner excitation than that derived with a dither piezo.

The research work presented in this paper aims to provide an algorithm that can correct parachuting in real time and allows faster scanning speeds, but with the novelty that it exploits only the standard AFM setup. In this way, the present strategy can be handily applied to any commercial AFM.

**Figure 3:**
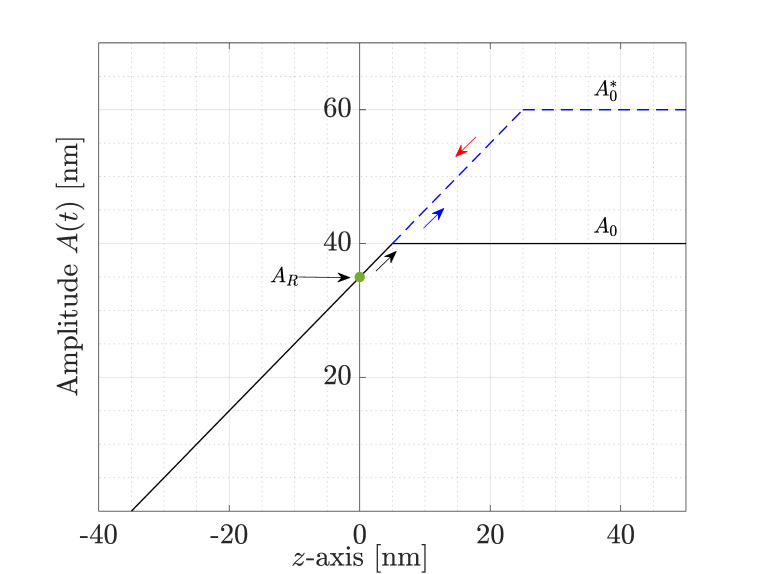
Dependence of the amplitude A(t) on the separation distance of the tip from the sample surface. The nominal situation is shown in black, while in blue, the proposed strategy to reduce parachuting shows an extended linear region.

In [13], the value $e_{sat}$ is increased by allowing a larger deflection y(t) at the parachuting phase, since $\varepsilon_{t}$ is inversely proportional to $e_{sat}$. In the standard setup of a DM-AFM, this can be accomplished by amplifying the excitation force $F_{exc}\left(t\right)$. In fact, once parachuting is started, (i.e., for e<0, see Figure 1), the interaction with the sample is lost, and the only force acting on the cantilever beam is the driving excitation $F_{exc}\left(t\right)$; see Equation (2). As a consequence, the beam will start oscillating with a free vibration amplitude $A_{0}$—easily deduced from the frequency response theorem—and the error will saturate to $e_{sat}=A_{R}-A_{0}$ eventually. Referring to Figure 3, this situation is depicted in black, with the black arrow pointing to the linear increment of A(t) from $A_{R}$ to $A_{0}$. However, if the excitation force is allowed to increase, then the free vibration amplitude will be larger as well. In fact, A(t) will move along the blue dashed line of Figure 3, as indicated by the blue arrow, until the new maximum value $A_{0}^{*}$ is reached. This, in turn, will lead to an increase in the saturation value of the error, hence shortening the parachuting duration. Then, once the interaction with the sample is restored, A(t) will decrease following the same straight line in the direction of the red arrow, until the set point $A_{R}$ is reached again.

The amplification of the driving force just described can be mathematically modeled as

(7) $$F_{exc}^{*}\left(t\right)=\left\{\begin{array}{cc} F_{exc}\left(t\right) & e(t)\geq 0\\ F_{exc}\left(t\right)G_{e}\left(t\right) & e(t)<0 \end{array}\right.$$

where $F_{exc}^{*}\left(t\right)$ is the new amplified driving force, and $G_{e}\left(t\right)>1$ is an error-dependent gain chosen as

(8) $$G_{e}\left(t\right)=1+\alpha \frac{e(t)}{e_{sat}}\;,\;\alpha >0.$$

**Figure 4:**
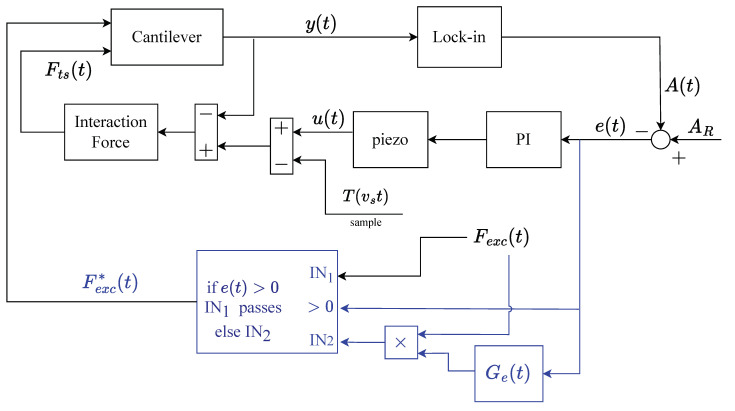
Block diagram of the Adaptive Drive (AD): in black is the standard DM setup, while in blue, the proposed “add-on” is shown.

The weight α>0 is an additional (adimensional) parameter to finely tune $G_{e}\left(t\right)$ during the experimental session. Basically, if α=0, the setup is identical to that of a DM-AFM, but if α>0, then a timely amplification is applied, and the more positive α is, the greater the correction. The modified control scheme, which will be referred to as Adaptive Drive (AD) since the driving force $F_{exc}\left(t\right)$ is dynamically adapted, is shown in Figure 4. There, the setup belonging to the standard DM is shown in black, while in blue, the proposed modifications are shown.

This approach, although very simple to implement, is actually very effective, as will be shown later on. Furthermore, despite the fact that it resembles the strategy of the Dynamic-PI controller [10], it does not amplify the noise in the loop. In fact, the bandwidth of the controller is left unchanged.

**Figure 5:**
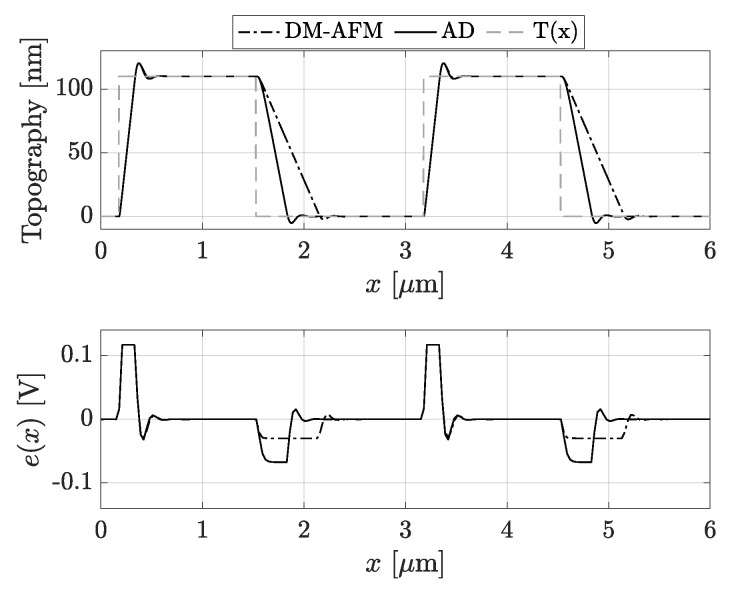
Comparison in a simulated environment of the traditional DM-AFM scheme (dot-dashed lines) and with the Adaptive Drive AD (continuous lines), either for the reconstructed topography $T_{r}\left(x\right)$ or the error e(x) in input to the controller. Clearly, the error $\varepsilon_{t}$ is significantly reduced in the reconstructed topography $T_{r}\left(x\right)$, while from the graph of e(x), it can be easily verified that the contact with the sample (inversion in the trend) is restored faster.

In Figure 5, two periods of a sample with a square-wave profile, T(x), are simulated, both with the traditional mode (dash-dot lines) and with the AD (continuous lines). In the topography graph, we can notice that the falling edges of AD are clearly steeper than those in DM, minimizing the error $\varepsilon_{t}$ and the parachuting phase. The latter can be seen by looking at the error graph $e(x=t/v_{s})$. In DM, e(x) saturates to $e_{sat}$, while the continuous line converges to a new value, denoted $e_{sat}^{*}$, with $e_{sat}^{*}>e_{sat}$, for a shorter time since the tip is brought back near the sample faster. Furthermore, this approach ideally allows the tip to never lose contact with the sample. In fact, if the applied gain $G_{e}\left(t\right)$ is large enough, the tip is driven to oscillate to a new free vibration amplitude $A_{0}^{*}$, such that the height of the slope $h_{0}<A_{0}^{*}-A_{R}$. Hence, parachuting will be immediately reset. Of course, the physical limits of the hardware must never be exceeded; therefore, if $h_{0}$ is too large, it will be impossible to nullify parachuting totally. Nevertheless, its negative effects will be definitely reduced.

### Implementation

Experiments were conducted on the DriveAFM from Nanosurf [19], a platform intentionally designed for research purposes, since it allows users to easily access output and input signals from and into the AFM. The measurements were taken on the NSG10Au cantilever beam by NT-MDT [20], with a resonance frequency $f_{n}$ of 160 kHz and a *Q* factor of 300 in the air.

The switching logic of Equation (7) was first rewritten in a more suitable way for the implementation. The AD mode algorithm is actually triggered not once e(t)<0 but on a specific (negative) threshold $e\left(t\right)<e_{th}$. When the threshold is suitably chosen, the adopted strategy not only makes the algorithm more robust to noise, since small fluctuations in e(t) do not activate the augmented forcing on and off, but also prevents stronger tip–sample interactions by anticipating the deactivation of the algorithm.

This algorithm has been implemented on an external FPGA connected to the DriveAFM. The FPGA was a MOKU PRO by Liquid Instruments [21] which allows users to easily code their custom functions, and change parameter values in real time. This feature mattered for testing different values of parameter α and then for choosing the one that best corrects parachuting.

**Figure 6:**
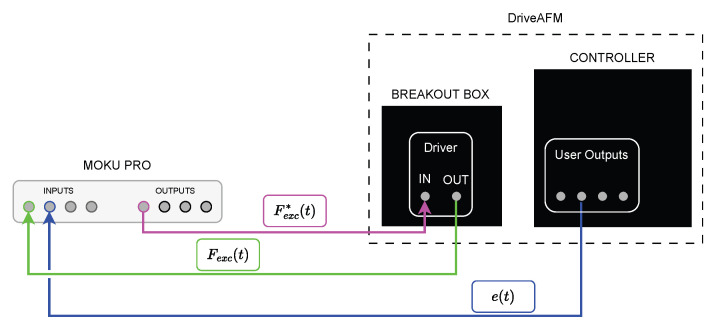
Schematic representation of the instrumentation connections, where the signals are defined in Equations (2), (3) and (7).

In Figure 6, a schematic representation is shown of the connections between the AFM and MOKU PRO. The DriverAFM comes equipped with the Controller box, which hosts the PI controller and allows reading signals internal to the controller—such as the error e(t)—and the breakout box. Additionally, the breakout box provides access to many signals, especially the driver excitation, which can be read and/or written.

MOKU PRO houses the algorithm, and through the aid of the Liquid Instruments App, downloadable on IPad or PC, it is possible to change α in real time; see [22,23].

Finally, experiments have been conducted over a standard grid calibration sample provided by Nanosurf, characterized by a pitch of 10 μm and a step height of 120 ± 4 nm. The excitation force $F_{exc}\left(t\right)$ was set at 100 mV, and the free amplitude $A_{0}$ and the set point $A_{R}$ were chosen as 30 nm and 75%$A_{0}$, respectively.

## 4. Experimental Results

**Figure 7:**
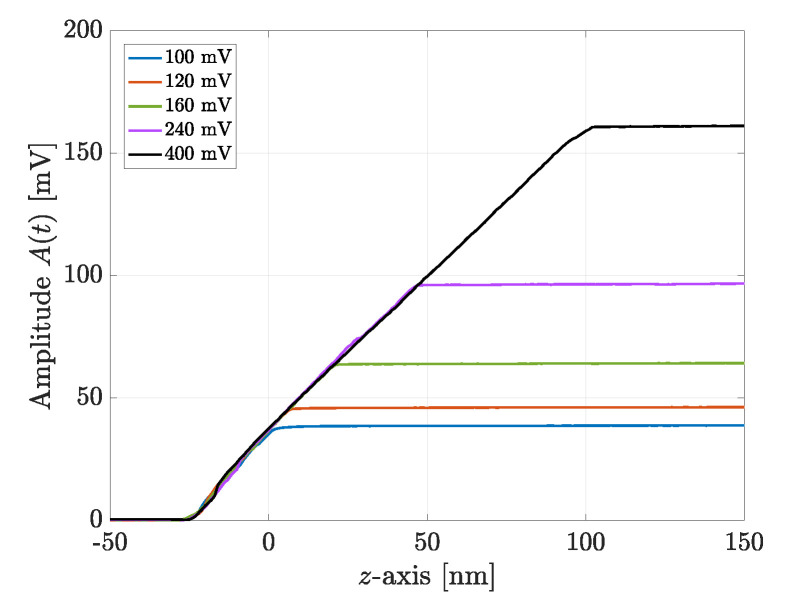
Set of amplitude–distance curves collected experimentally for several driving forces, and hence free amplitudes, to demonstrate the same exact linear growth for each case.

The first experiments aimed to verify the hypothesis that the tip–sample distance curve does not change for different values of the driving force. In other words, in relation to Figure 3, the objective was to check that the slopes of the black and blue curves were the same. The amplitude–distance curves were generated for five different values of the driving force within the interval [100, 400] mV, and the results are shown in Figure 7. All the straight lines in the contact region maintain the same slope. These results guarantee that the AD algorithm does not introduce any artifacts.

Moreover, Figure 7 also shows that the free amplitude is proportional to the driving force, confirming that the cantilever beam behaves as a linear system.

**Figure 8:**
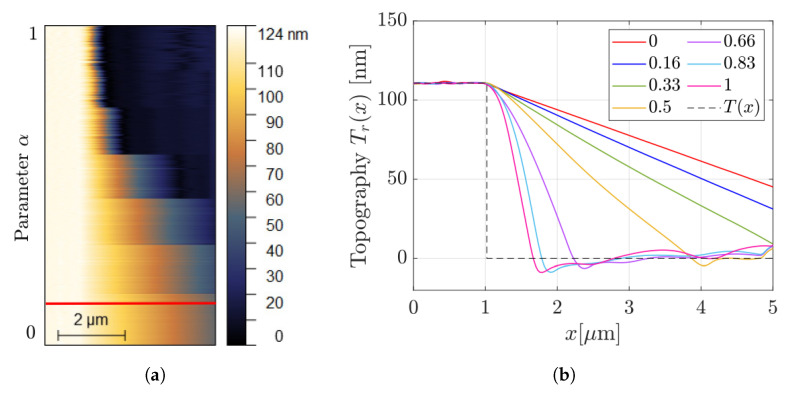
(**a**) Multiple scans of the same line across a descending step (scanning direction from left to right) for seven different values of α, evenly spaced in the interval [0, 1]. To enhance data comprehension, in (**b**), profiles are drawn for each α step of (**a**).

The subsequent experiments were designed to test the performance of AD for different values of the parameter α in Equation (8). Repeated scans over the same line in correspondence to a falling edge were performed at constant speed and changing only the parameter α. The outcomes are shown in Figure 8a, where the parachuting duration is reported for growing values of α∈[0, 1] starting from the bottom, as indicated by the vertical axis. The same kind of information is then given on the *x*-*y* plane (Figure 8b), where $T_{r}\left(x\right)$ has been extracted from one profile for each α value in Figure 8a. As an example, the red curve of Figure 8b corresponds to the profile taken on the red line of Figure 8a. For growing values of α, as expected from Section 3, $\varepsilon_{t}$ is gradually reduced.

**Figure 9:**
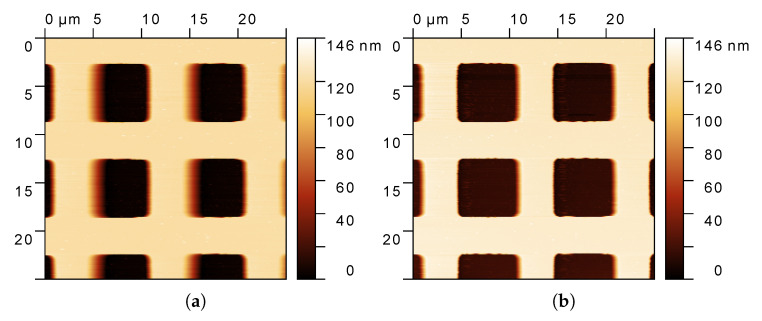
Pseudo-color maps of a grid calibration sample HS-100MG (Tedpella) with a step height 120 ± 4 nm scanned at $v_{s}=100$ μm/s from left to right. (**a**) The map generated with the standard DM. (**b**) The map generated with the proposed AD algorithm. Benefits are clear on the falling edges; the blurred regions (parachuting) are strongly reduced by AD.

In order to better appreciate the benefits of the algorithm, the pseudo-color map $T_{r}\left(x,y\right)$ has been collected over a scanning area of 25 × 25 μ$m^{2}$, and compared with that generated using DM; see Figure 9. With the scanning direction from left to right, the effects of the parachuting are evident in Figure 9a (DM) on the falling edges of the holes. In fact, those edges appear to be blurred due to the slow action of the controller, causing a wrong assessment of the real height in those locations of the sample. On the opposite, the same profiles are sharp and clean in Figure 9b, in which AD is running, proving the validity of the proposed strategy.

**Figure 10:**
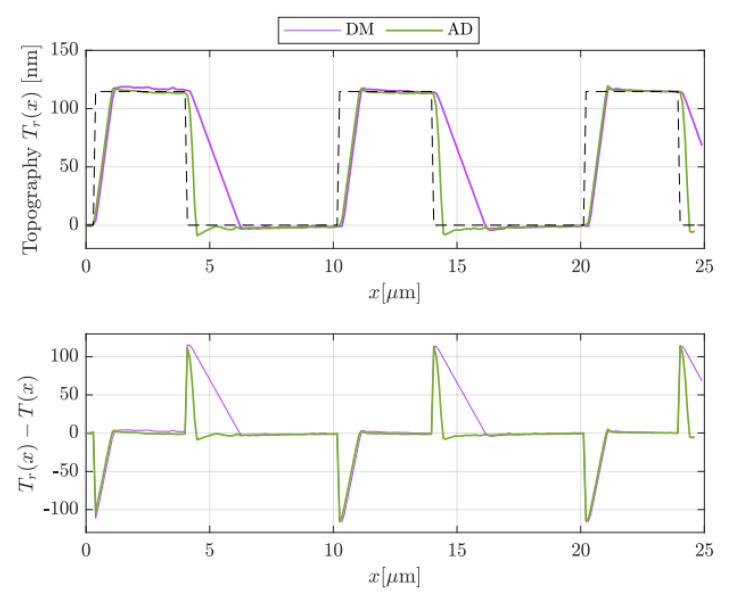
On **top**, the profiles taken over five consecutive scanning rows of Figure 9a (purple curve), and Figure 9b, green curve, along with the reference topography T(x) (dashed line). On the **bottom** graph, the difference between the reconstructed and reference topographies.

A more quantitative evaluation of the improved performances of AD with respect to DM is reported in Figure 10. There, the reconstructed topographies of Figure 9a,b are depicted in the upper graph in purple and green, respectively. The reference topography T(x) (dashed line)—computed on the basis of the datasheet—has been drawn for a better comparison between DM and AD modes. Afterwards, the topography errors, meant as the difference between the reconstructed and the reference topographies, i.e., DM −T(x) and AD −T(x), are depicted in the lower graph of Figure 10. There, the positive and negative peaks occur in correspondence to abrupt variations in the sample topography, which the feedback loop is not able to track properly. From both graphs of Figure 10, the benefits of AD mode are evident, since the parachuting is clearly recovered earlier. However, this may occur at increased interaction forces due to a larger tip oscillation amplitude. In this regard, the green curve clearly exhibits a more accentuated undershoot once the tip is brought back to the sample. Nevertheless, in AD mode, the mean tip–sample interaction force could be set lower with respect to standard DM. In fact, being able to strongly reduce parachuting, AD allows users to choose a set point amplitude $A_{R}$ much closer to the free oscillation amplitude $A_{0}$.

**Figure 11:**
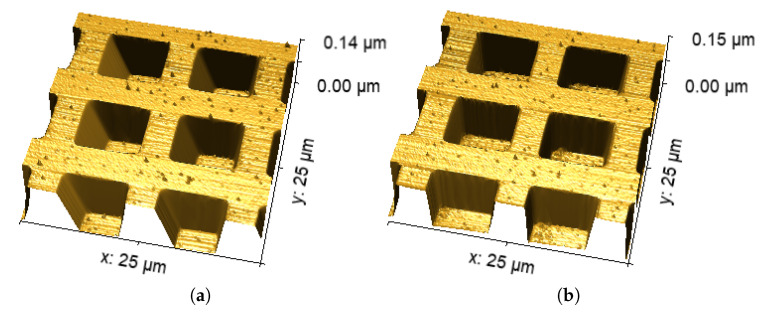
Three-dimensional view reconstruction of the calibration grating, without (**a**) and with (**b**) the active algorithm (MOKU ON).

Finally, for the sake of clarity, in Figure 11, the corresponding 3D reconstructions of Figure 9a,b are shown. Clearly, in Figure 11a (algorithm Off), error $\varepsilon_{t}$ is much greater than that of Figure 11b (algorithm On).

**Figure 12:**
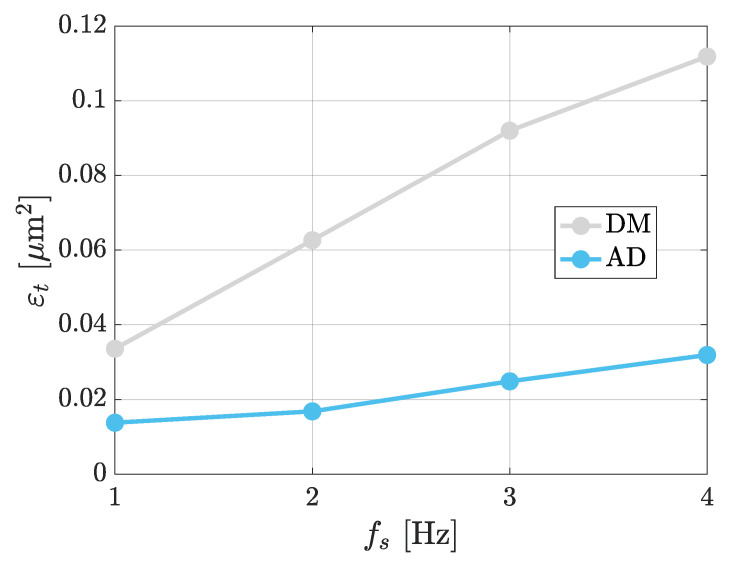
Comparison of error $\varepsilon_{t}$ in the reconstructed topography using either the traditional DM (gray) or AD (blue) at speeds $f_{s}=1$ Hz, 2 Hz, 3 Hz and 4 Hz.

The objective of this work was to develop a method for reducing parachuting, but at the same time allow for fast scanning speeds. For testing this latter point, experiments were still conducted on the same grating sample, but at different scanning frequencies between 1 Hz and 4 Hz. For the case with the algorithm active, the value of α was fixed for all trials in order to better assess how speed impacts the performances. Therefore, assuming α∈[0,1], the used value for each test was α=1 in order to obtain the best performance at the lowest speed (1 Hz). The comparison between the standard approach and AD is shown in Figure 12. The chosen rule to compare was to evaluate the error $\varepsilon_{t}$ at the different speeds. The gray line represents the errors in the reconstructed topography for DM, while in blue are shown those for the AD strategy (MOKU On). The improvements are evident, as the blue line is always below the gray one, and such improvements become more evident as the frequency (and hence the speed) is increased; see the cases at 3 Hz and 4 Hz. Furthermore, notice that the error $\varepsilon_{t}$ at 4 Hz with AD matches the one at 1 Hz obtained with the standard DM. Hence, it turns out that the proposed algorithm allows scanning frequencies up to four times faster than those with the standard mode.

## 5. Conclusions

In this paper, taking inspiration from a previous study, a new correction on the standard feedback controller of DM-AFMs has been suggested in order to overcome the typical trade-off between fast scanning speeds, high accuracy, and low interaction forces. In particular, the developed algorithm—Adaptive Drive (AD)—aims to mitigate parachuting, an undesired effect which arises at steep falling edges of the sample and causes artifacts in the reconstructed topography.

The proposed technique is easily implemented on standard DM-AFMs, since it is not necessary to make direct changes to the original control scheme, and the hardware implementation can be realized with an external device having access to some signals. Specifically, the strategy pushes the cantilever beam to oscillate with a larger free vibration amplitude. In this way, a major error signal is fed to the controller, which in turn reacts faster and reduces the parachuting artifacts while ensuring low interaction forces.

To validate the AD method, the effectiveness of the algorithm has been tested on calibration samples. The experiments clearly show the sharp improvements reached with AD with respect to the standard DM. In fact, the error in the reconstructed topography is greatly reduced. Furthermore, tests prove that the method also reduces the scanning time, since it allows up to four times faster scanning speeds. In conclusion, the work discussed in this paper paves the way for new possible AFM controllers, which ensure better imaging sessions by reducing the acquisition time and improving accuracy while maintaining low interaction forces. To enhance the strength of AD, specific tests on biological samples are planned and will be treated in a separate work. 

## Data Availability

The data presented in this study are available on request from the corresponding author.

## Abbreviations

The following abbreviations are used in this manuscript:

AFM	Atomic Force Microscope
DM	Dynamic Mode
AD	Adaptive Drive
